# Robotic arm-assisted unicondylar knee arthroplasty resulted in superior radiological accuracy: a propensity score-matched analysis

**DOI:** 10.1186/s42836-023-00210-6

**Published:** 2023-11-02

**Authors:** Matthew H. Y. Yeung, Henry Fu, Amy Cheung, Vincent Chan Wai Kwan, Man Hong Cheung, Ping Keung Chan, Kwong Yuen Chiu, Chun Hoi Yan

**Affiliations:** 1 Li Ka Shing Faculty of Medicine, School of Clinical Medicine, The University of Hong Kong, Hong Kong SAR, China; 2 Department of Orthopaedics and Traumatology, School of Clinical Medicine, The University of Hong Kong, Hong Kong SAR, China; 3 Department of Orthopaedics and Traumatology, Queen Mary Hospital, Hong Kong SAR, China; 4 Department of Orthopaedics and Traumatology, Gleneagles Hospital Hong Kong, Hong Kong SAR, China

**Keywords:** Unicompartmental knee arthroplasty, Robotic surgery, Knee replacement, Partial knee replacement, Robotic arm-assisted knee replacement

## Abstract

**Introduction:**

Unicompartmental knee arthroplasty (UKA) is an effective surgical treatment for medial compartment arthritis of the knee, yet surgical outcomes are directly related to surgical execution. Robotic arm-assisted surgery aims to address these difficulties by allowing for detailed preoperative planning, real-time intraoperative assessment and haptic-controlled bone removal. This study aimed to compare the clinical and radiological outcomes between conventional manual mobile bearing and robot arm-assisted fixed bearing medial UKA in our local population.

**Materials and methods:**

This is a retrospective case–control study of 148 UKAs performed at an academic institution with a minimum of 1-year follow-up. 74 robotic arm-assisted UKAs were matched to 74 conventional UKAs via propensity score matching. Radiological outcomes included postoperative mechanical axis and individual component alignment. Clinical parameters included a range of motion, Knee Society knee score and functional assessment taken before, 6 and 12 months after the operation.

**Results:**

Robot arm-assisted UKA produced a more neutral component coronal alignment in both femoral component (robotic -0.2 ± 2.8, manual 2.6 ± 2.3; *P* = 0.043) and tibial component (robotic -0.3 ± 4.0, manual 1.7 ± 5.3; *P* < 0.001). While the postoperative mechanical axis was comparable, robot arm-assisted UKA demonstrated a smaller posterior tibial slope (robotic 5.7 ± 2.7, manual 8.2 ± 3.3; *P* = 0.02). Clinical outcomes did not show any statistically significant differences.

**Conclusion:**

Compared with conventional UKA, robotic arm-assisted UKA demonstrated improved component alignment and comparable clinical outcomes. Improved radiological accuracy with robotic-arm assistance demonstrated promising early results.

## Introduction

The prevalence of osteoarthritis is on the rise in recent years [1, 2]. Krutz et al. suggested the demand for knee replacement will increase by more than sixfold by 2030 [3]. Unicompartmental knee arthroplasty (UKA) is an effective surgical treatment for medial knee osteoarthritis [4], with less surgical trauma and faster recovery. However, its use has not been widely popularized due to its higher revision rate, with surgical outcomes directly related to surgical technique. Collier et al. [5] revealed prosthesis malalignment can lead to early failure of UKA and is likely to contribute to the higher revision rate observed with UKA as compared with TKA (1.4% vs. 4.6% at 3 years). Hiranaka et al. [6] showed an increased risk of tibial fracture post UKA in Asian patients compared to Caucasians, likely due to their difference in bone size and anatomy. The same study outlined a radiological parameter that predicted such fracture risk, further stressing the importance of component radiological accuracy in preventing post-UKA fractures. With the introduction of the robotic arm-assisted system in 2006, intraoperative alignment can be assessed in real-time, prior studies have also demonstrated that robot arm-assisted UKA could achieve improved implant positioning over manual instrumentation [7–9]. Robotic-assisted joint replacement surgery was first utilized in public hospitals in Hong Kong in 2019 upon approval by Hospital Authorities Mechanism for the Safe Introduction of New Procedure/Technology (*HAMSINP*) for two robotic systems, namely, Bluebelt Navio (Smith and Nephew, Memphis, TN, USA), an imageless robotic system and the Mako robotic arm system (Stryker, Fort Lauderdale, FL, USA), an image-based robotic system. This study aimed to compare the early clinical and radiological outcomes between conventional manual, mobile-bearing medial UKA and robot arm-assisted, fixed-bearing medial UKA. We hypothesized that robot arm-assisted UKA would result in superior outcomes.

## Materials and methods

### Study design

**Fig. 1:**
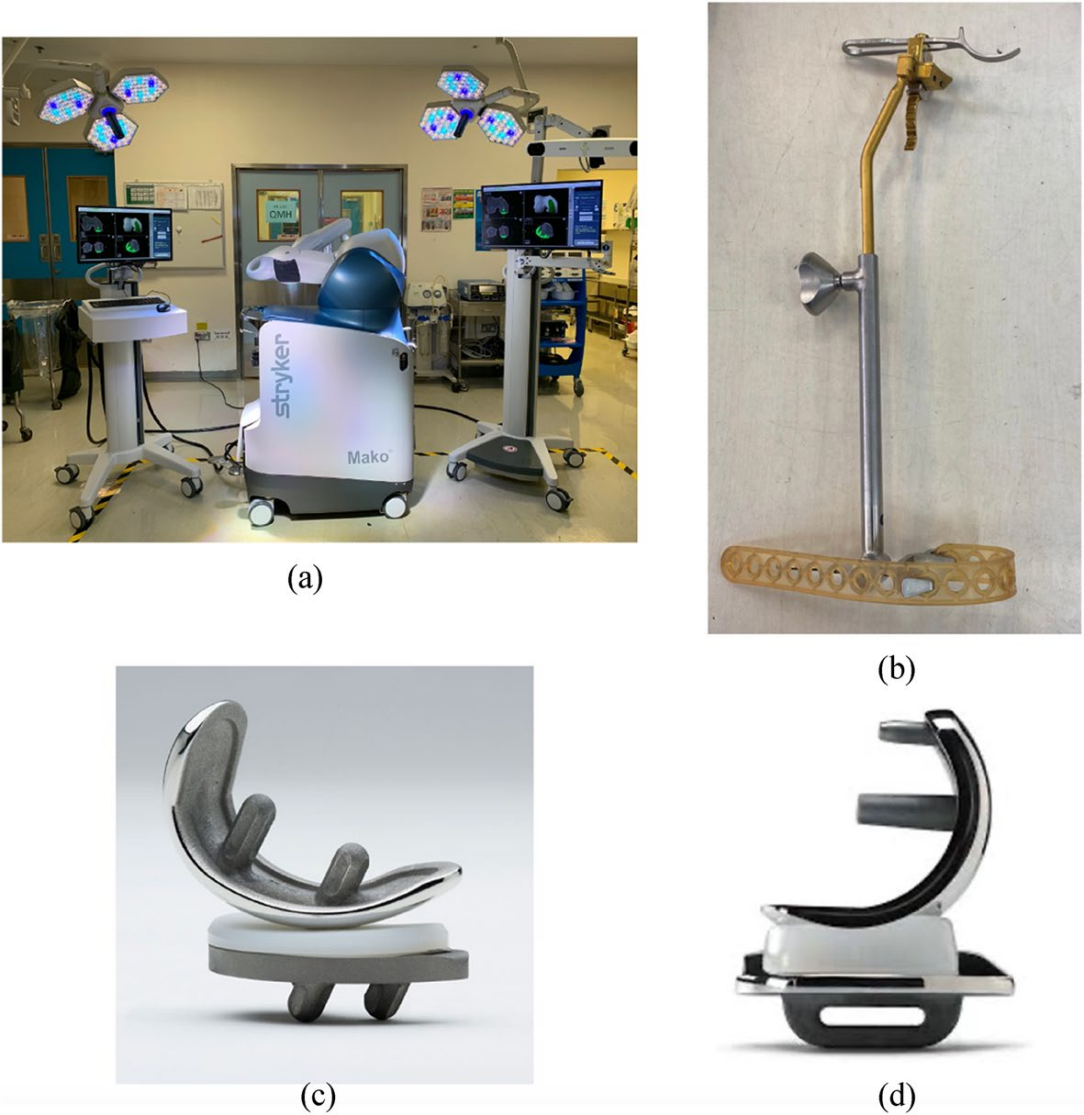
Conventional and robotic techniques and implants

This is a retrospective case–control study to compare clinical and radiological outcomes between robotic arm-assisted UKA and conventional jig-based UKA (Fig. 1). The study was conducted by following published checklists Strengthening the Reporting of Observational Studies in Epidemiology (STROBE) for cohort studies.

**Fig. 2:**
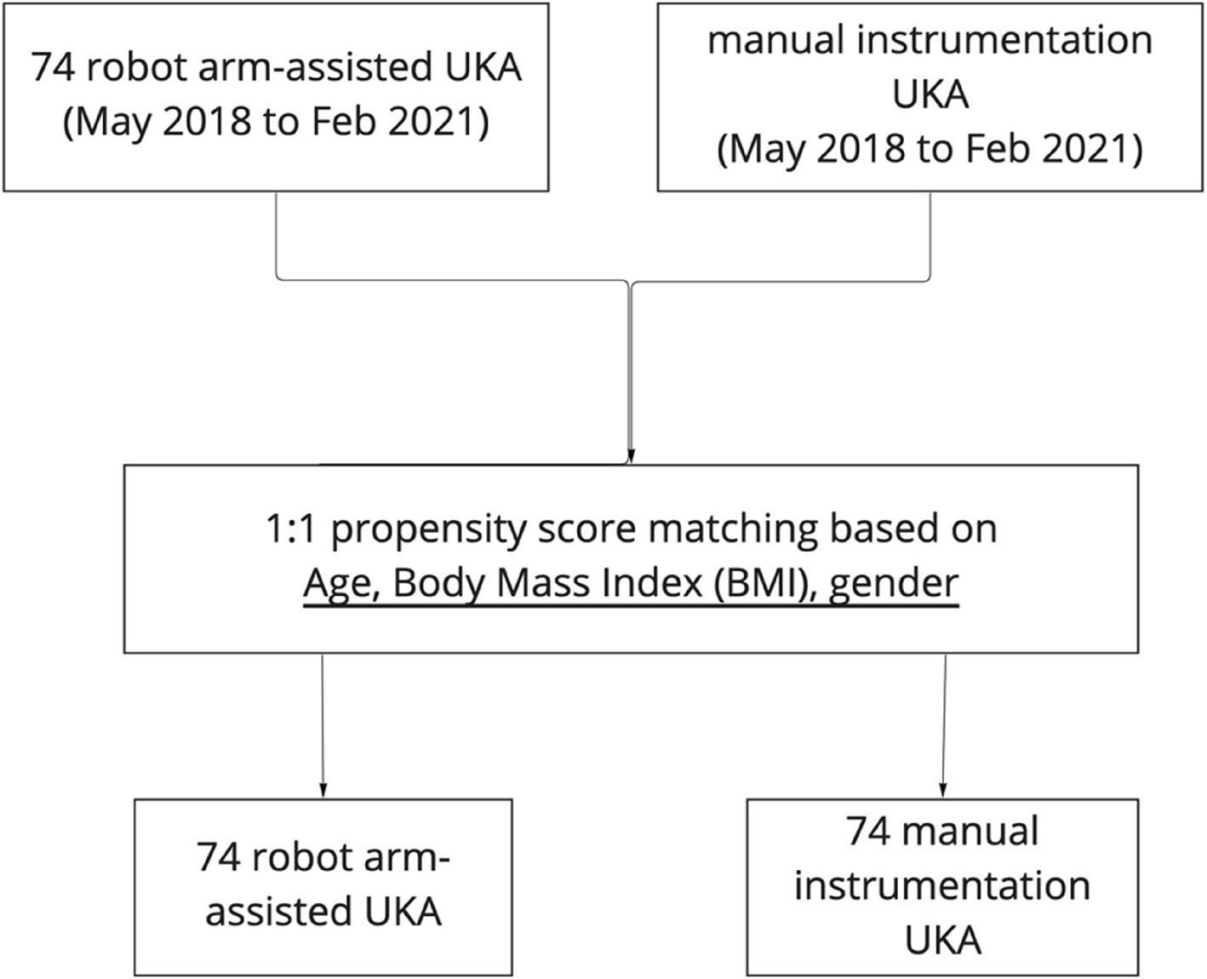
Flowchart for cohort

Seventy-four knees from 70 patients who had undergone robotic arm-assisted UKA were analyzed in a single academic institution between May 2018 to Feb 2021, with a mean follow-up period of 18.1 ± 6.1 months. Patients with bilateral UKA were not excluded, with each knee counted as an independent entry. To limit bias and confounding factors such as baseline differences of patients, the robotic-assisted UKAs were matched with manual instrumentation UKAs done in the same institution over the same time period at a 1:1 ratio using propensity score matching (PSM) based on nearest neighbor matching without replacement within a caliper width of 0.2 (Fig. 2). Parameters chosen for inclusion in PSM calculation included age, Body Mass Index (BMI), and gender. The standard mean differences (SMD) were calculated to evaluate the balance of covariates between the two surgical groups. The final PSM calculation had a total of 148 knees from 140 patients enrolled in the final analysis, yielding 74 cases for each of the surgical techniques.

Inclusion criteria included patients diagnosed with isolated medial compartment arthritis or osteonecrosis of the medial femoral condyle who had undergone medial UKA with a minimum 1-year follow-up. Patients with non-medial UKA, bicompartmental or tricompartmental knee replacements were excluded.

Given the retrospective nature of this study, certain data loss is expected. Multiple imputation was performed to handle these missing data.

### Surgical techniques

The Mako robotic arm system (Stryker, Fort Lauderdale, Florida, USA) was used in our study. Individual preoperative computer tomography (CT) scans were performed for surgical planning. The scans were segmented and developed into a 3-dimensional knee model allowing for preoperative implant planning. The process was individualized to minimize bone resection and restore native peri-arthritic joint anatomy. Femoral and tibial components were planned neutral to the mechanical axis. The posterior slope was planned to match the patient’s native anatomy, ranging from 3° to 7°. Lower limb alignment targeted for under-correction, ranging between 3° to 7°. Intraoperatively, reflective marker arrays were positioned on the tibia and femur using stab incisions, registration of anatomical landmarks by using optical motion capture technology and a registration probe. This mapped the surgical field and allowed for dynamic referencing of the tibia and femur for the final orientation of the implants on the 3D model. The robotic arm allowed for accurate intraoperative resection using high-speed water-cooled burr and imaging of 3-dimensional boundaries. The robotic arm provides tactile, visual and audio feedback upon resection. In the rare case, where the burr had to go beyond the predetermined boundary, the robotic arm would shut down. MAKOplasty UKA 2.5 software was used for the blur only. The implant used was Stryker Restoris MCK, consisting of a cobalt chrome femoral component, and a titanium tibial component with a fixed-bearing polyethylene insert.

For manual instrumentation, Oxford phase 3 microplasty instruments were used. Femoral and tibial components were planned and templated to achieve neutral coronal alignment, while the tibial slope target was 7°. The limb alignment target was under-corrected into varus between 3°–7°, depending on the degree of preoperative deformity. Following a medial parapatellar approach, osteophytes were first removed and the medial meniscus excised while protecting the medial collateral ligament. The verticalcal tibial cut was performed using a hand-held saw guided by a cutting jig. Horizontal tibial cut was performed using a hand-held oscillating saw. A hole in the intramedullary canal of the femur was drilled for the insertion of femoral drill guide. After confirming alignment, the posterior femoral cut was made using a chisel. The distal femur was then milled and flexion/extension gaps were equalized. Finally, the tibial plateau was prepared, components were cemented, and polyethylene mobile bearing of appropriate thickness was inserted to ensure adequate soft tissue tension. Release of the medial collateral ligament was not performed.

### Outcome measures

**Fig. 3:**
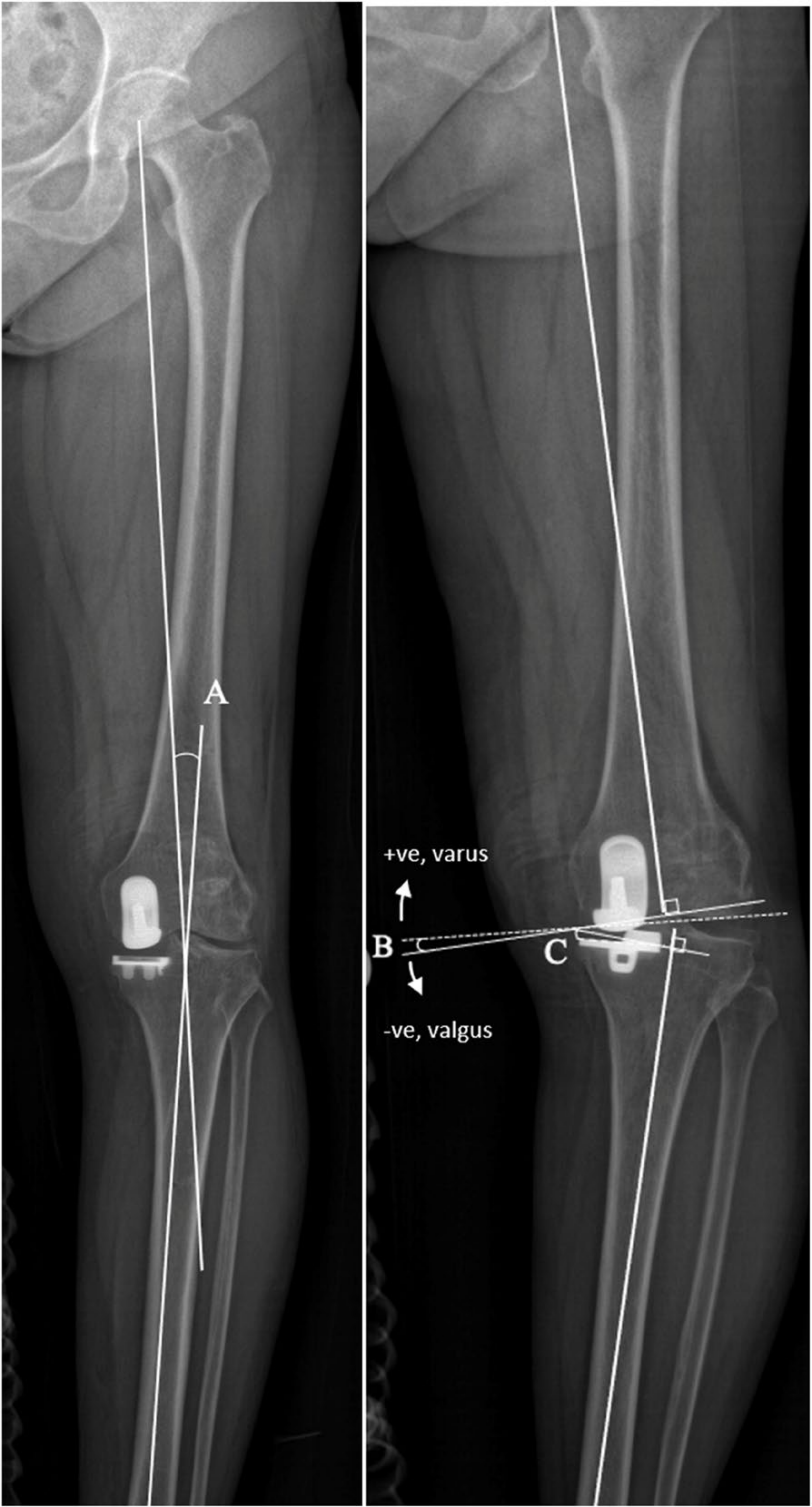
Coronal film measurements

**Fig. 4:**
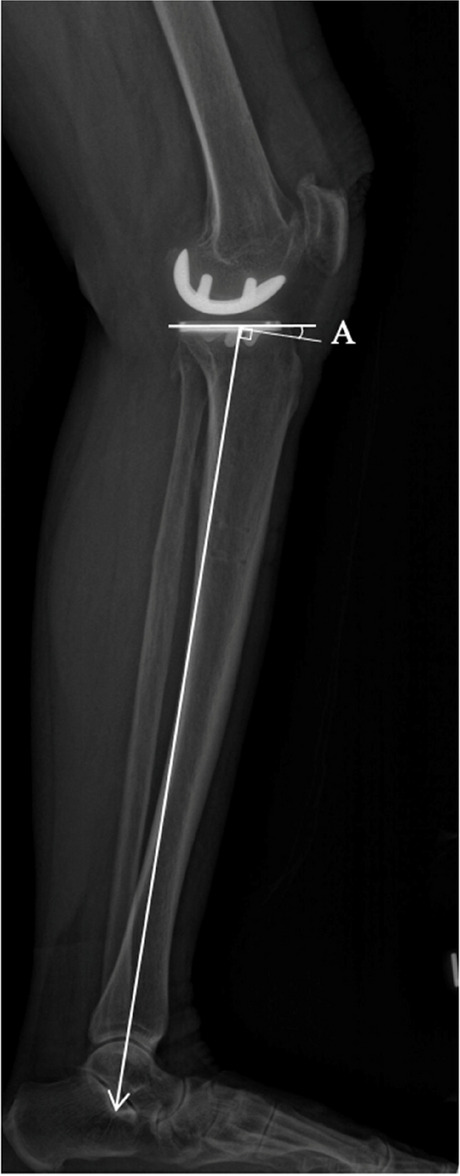
Sagittal film measurements

In this paper, we reported the radiological and clinical outcomes with radiological outcome as the primary outcome measure. Radiological outcomes include postoperative lower limb mechanical alignment, tibial component (coronal and sagittal alignment), and femoral component (coronal alignment), on the basis of long-standing, anteroposterior and lateral films of the lower limb. In the coronal view (Fig. 3), the mechanical axis measures the Hip-Knee-Ankle (HKA) angle (Fig. 3, angle A), i.e., the angle formed by the mechanical axis of the femur and the mechanical axis of the tibia. Femoral component coronal alignment was attained by measuring the angle between the distal femur cut surface and the mechanical axis of the femur (Fig. 3, angle B). Tibial component alignment was obtained by measuring the angle formed by the proximal border of the tibial baseplate and the mechanical axis of the tibia (Fig. 3, angle C). In the sagittal view (Fig. 4), the posterior slope was obtained by measuring the angle between the tibial baseplate and the line between the tibial component and the line between the centre of the ankle and centre of the knee on the long film (Fig. 4, angle A).

Clinical outcomes include Knee Society knee score (KSKS), Knee Society functional assessment (KSFA), and range of motion (ROM), measured by physiotherapists in our clinic using a goniometer separately before, 6 months and 12 months after operation.

### Statistical analysis

Statistical analysis was done using Statistical Package for the Social Sciences (SPSS), ver 28.0 (Hong Kong), plus the software package R plugin. Logistic regression analysis was used to examine the association between the type of UKA and outcomes for propensity score matching. Given the normal distribution nature of our data, independent samples *t*-tests were used to determine whether the difference of continuous variables in each of the surgical outcomes was statistically significant. The normal distribution of data was determined using the Shaprio-Wilk test performed in SPSS. The chi-square test was employed to compare categorical data. In the cases of missing data, multiple imputations in statistical software R were used. All Statistical significance was defined as a *P-*value of 0.05.

## Results

### Radiological outcomes

We report postoperative radiological measurements in Table 1. Robot arm-assisted UKA produced more neutral femoral and tibial component coronal alignments. For the femoral component, the coronal alignment of the robotic arm was valgus 0.2° ± 2.8°, compared to varus 2.6° ± 2.3° of manual instrumentation (*P* < 0.001). For the tibial component, the alignment of the robotic arm was valgus 0.3° ± 4.0°, compared to varus 1.7° ± 5.3° of manual instrumentation (*P* = 0.009). Robot arm-assisted UKA demonstrated a less posterior tibial slope than manual instrumentation (5.7° ± 2.7° vs. 8.2° ± 3.3°) (*P* < 0.001). Looking at the whole limb, the postoperative mechanical axis was not statistically different between the two surgical techniques, being 4.4° and 4.8° for robotic-arm assisted and manual instrumentation respectively (*P* = 0.750).

**Table 1:** Radiological outcomes

	Robot arm-assisted (*n* = 74)	Manual instrumentation (*n* = 74)	*P-*value
Posterior tibial slope (degree)	5.7 ± 2.7	8.2 ± 3.3	< 0.001*
Tibial component alignment (degree)	-0.3 ± 4.0	1.7 ± 5.3	0.009*
Femoral component alignment (degree)	-0.2 ± 2.8	2.6 ± 2.3	< 0.001*
Lower limb alignment (degree)	4.4 ± 3.9	4.8 ± 3.3	0.306

^*^statistically significant, *P* ≤ 0.05

### Clinical outcomes

Clinical outcomes were recorded before, 6 and 12 months after operation, including Knee Society knee score (KSKS), and Knee Society functional assessment (KSFA) (Table 2). For robotic arm-assisted UKA, KSFA was 52.3 ± 12.6, 61.8 ± 15.7, 71.5 ± 14.7 before, 6 and 12 months after operation, respectively, against 53.8 ± 13.7, 66.0 ± 17.6, 79.2 ± 36.5 with manual instrumentation during the same time frame. KSKS with robotic arm-assisted UKA was 50.5 ± 9.4, 89.3 ± 11.3, 92.2 ± 5.6 before, 6 and 12 months after operation. During the same time period KSKS was 54.2 ± 8.9, 90.7 ± 9.5, 93.1 ± 8.0 with manual instrumentation. There was no statistically significant difference between all scores across all time periods, and improvement was found in both clinical scores with time in both surgical techniques. Additional parameters of clinical outcomes recorded were the knee’s range of motion (ROM), and the results were also similar. ROM for robotic-arm assisted UKA was 113.2° ± 10.3°, 112.1° ± 10.8°, 113.9° ± 11.4° before, 6 and 12 months after operation, respectively against 112.5° ± 13.3°, 109.6° ± 11.1°, 112.9° ± 11.0°, respectively, with manual instrumentation.

**Table 2:** Secondary clinical outcomes

	Robot arm-assisted (*n* = 74)	Manual instrumentation (*n* = 74)	*P*-value
Knee Society Functional Assessment (KSFA) Pre-Operative	52.2 ± 12.6	53.8 ± 13.7	0.241
Knee Society Functional Assessment (KSFA) 6 months postoperatively	61.8 ± 15.7	66.0 ± 17.6	0.096
Knee Society Functional Assessment (KSFA) 12 months postoperatively	71.5 ± 14.7	79.0 ± 36.5	0.127
Knee Society Knee Score (KSKS) Preoperatively	50.5 ± 9.4	54.2 ± 8.9	0.014
Knee Society Knee Score (KSKS) 6 months postoperatively	89.3 ± 11.3	90.7 ± 9.5	0.242
Knee Society Knee Score (KSKS) 12 months	92.2 ± 5.6	93.1 ± 8.0	0.283
Range of Motion (ROM) (Flexion–Extension) Preoperatively	113.2 ± 10.3	112.5 ± 13.3	0.394
Range of Motion (ROM) 6 months postoperatively	112.1 ± 10.8	109.6 ± 11.1	0.156
Range of Motion (ROM) 12 months postoperatively	113.9 ± 11.4	112.9 ± 11.0	0.362

### Surgical outcomes

The mean follow-up period lasted for 18.1 ± 6.1 months with no loss to follow-up in either group. Implant survivorship was comparable. There were 3 revision cases of robotic-arm assisted UKA and 1 revision case for manual instrumentation UKA but the difference was not statistically significant (*P* = 0.945). All 4 revision cases of UKA were due to tibial component loosening.

Operation time, defined as the time of period from application to removal of the tourniquet on the surgical table, was measured. The mean operation time was 93.0 ± 20.6 min for robot arm-assisted UKA, and 101.4 ± 36.8 min for manual instrumentation (*P* = 0.097).

## Discussion

This is the first study to compare conventional and robotic unicompartmental knee arthroplasty in Hong Kong. Over the years, we have found mounting evidence of racial differences in orthometric measurements between Asians and Caucasians [10–12]. In addition, the dimensions of Chinese knees are shown to be generally smaller than the white knees, with Chinese females having a significantly narrower distal femur, and Chinese males having a wider proximal tibia than their white counterparts [13]. Smaller knees imply smaller components and a smaller surface area comes with an increased risk of aseptic loosening. Therefore, a more accurate individual component alignment is needed.

Robotic techniques have been implemented to improve implant accuracy and enhance postoperative rehabilitation [14] and its superior implant precision has been widely demonstrated across multiple different robotic platforms [15–18]. The robotic arm system employs three-dimensional (3D) imaging reconstruction technology based on preoperative and intraoperative computed tomography (CT) scans, as an attempt to improve bone cut accuracy [19]. Our primary outcomes in terms of improvement in both individual component position and lower limb alignment in robotic arm-assisted UKA were consistent with other studies [12–14]. There is growing evidence that individual component alignment and lower limb alignment are major factors dictating survivorship of UKA [20–22], and the result, if further validated, could justify the slightly increased cost of robotic arm-assisted UKA.

Our study indicated that robotic arm-assisted UKA produced a significantly smaller posterior tibial slope compared to manual instrumentation UKA (5.8° ± 2.9°, 8.6° ± 3.4°, *P* < 0.001). A posterior tibial slope of 5°–10° has been frequently quoted as normal and increased as the knee degenerates. Chiu et al. [11] have reported a larger posterior slope than 2°–3° in the Caucasian population. A posterior tibial slope out of the “normal” range has been found to correlate with various pathologies. An excess posterior tibial slope could increase the articular cartilage contact stress [23], leading to the accelerated degeneration of unsurfaced lateral compartment. Hernigou et al. [24] have demonstrated that a posterior tibial slope > 7° could affect the sagittal displacement in a knee arthroplasty. All patients in their cohort with a posterior tibial slope > 10° were reported to develop anterior cruciate ligament rupture post UKA, a complication with a prevalence of 6.2%. The same patients had had increasing anterior translation since 1 year after operation, suggesting that the anterior cruciate ligament disruption is likely due to increased sagittal slope of the tibial component. In our study cohort, none of the robot-arm assisted patients had a postoperative posterior tibial slope of > 10°, meaning the risk of anterior cruciate ligament rupture is less compared to the manual instrumentation cohort.

Component alignment has long been proven to be vital in achieving optimal clinical outcomes in UKA, including improvement in knee score [25], range of motion (ROM) [26] and implant survivorship [24]. Yong et al. [25] demonstrated a significant two-way interaction between tibial component coronal angle and femoral component coronal angle that helped achieve significant survival benefit at 15 years, and that a 2°–4°of tibial component coronal angle led to the greatest long-term improvement in knee scores and implant survivorship. This finding that deliberate under-correction of tibial coronal alignment meant less transferred load to the lateral compartment of the knee has also been shown by Innocenti et al. [27], with an extended UKA survivorship. The same relationship has been demonstrated by multiple studies in other regions, studies by Swienckowski and Page [28] and Sekiguchi et al. [29], both concluded that a 2°–3° varus of tibial component resulted in superior clinical outcomes.

Our robot-arm assisted cohort fell under Yong’s “optimal group” of the tibial component coronal angle of 2°–4° and femoral component coronal angle of 0°–3° [25], corresponding to a 100% 15-year survivorship in his cohort. A statistically significant more varus coronal alignment of the femoral component was achieved in our manual instrumentation cohort, but the difference was not clinically significant. A future long-term review may show otherwise.

We measured the mechanical axis using whole lower limb standing radiographs, which yielded improved reliability of the measurement compared to short knee radiographs commonly used in other studies. There was no statistically significant difference between the two surgical techniques. Our whole lower limb data were concurrent with the increased lower extremity varus in the Asian population previously reported by Tang et al. [10, 30] compared to Caucasians. This might contribute to the increased ratio of knee osteoarthritis to hip osteoarthritis (9:1 in China, 3:1 in the USA) [10].

This paper presented the clinical outcomes and implant survivorship as our secondary outcome. Our follow-ups showed that there were no clinically or statistically significant differences between the groups at 1 year, in terms of KSFA, KSFS, and ROM. This is expected to result from the clinically insignificant difference in alignment. Our revision rate was comparable between the two surgical techniques, and the rate was similar to the previous prospective multicentre study conducted by Pearle et al. [31]. This indicates that almost all of our patients achieved good results postoperatively, but we could not differentiate the good and excellent outcomes between the two surgical techniques. This is consistent with a previous randomized control trial by Gilmor et al. [32].

This study’s strength was the minimal drop-out rate at one-year postoperative follow-up of all 148 patients in one high-volume institution, as well as a study exclusively involving Asian population. Limitations included that this was a single observer study, comparison between fixed bearing and mobile bearing implants and limitations encountered with radiographs. Robotic arm-assisted, fixed-bearing UKA implant often requires more precise alignment due to their round-on flat articulation, theoretically causing uneven weight loading on the edges due to a potential contact point [33]. Whereas with manual instrumentation, mobile bearing provides a spherical femoral component on a congruent insert, evenly distributing stress over the tibial component for weight bearing. We could not remove this confounding variable due to the separate bearing design of the two surgical techniques. Lastly, although we corrected baseline differences between the two groups of patients through propensity scores, bias remained. Further research involving higher levels of clinical evidence with longer follow-ups and large sample size is needed to further validate our findings.

While we demonstrated increased component accuracy, clinical scores remained similar, and it is unclear whether this translates into improved implant survivorship. Further follow-up of the study cohort is required to determine if the result remains valid in the longer run.

## Conclusion

In this study, robotic arm-assisted UKA demonstrated a higher component accuracy compared to conventional UKA, with no difference found in complication rate and superiority in patient-reported outcomes 1-year post-operation. The long-term outcomes of robotic arm-assisted UKA are still unclear compared to that of conventional UKA, and further studies are needed to further justify its cost.

## Data Availability

The datasets generated and/or analyzed during the current study are not publicly available due to patient privacy but are available from the corresponding author upon reasonable request.

## Abbreviations

UKA: Unicompartmental knee arthroplasty
TKA: Total knee arthroplasty
PSM: Propensity score matching
SMD: Standard mean differences
KSKS: Knee Society knee Score
KSFA: Knee Society functional assessment
ROM: Range of motion
SPSS: Statistical Package for the Social Sciences
3D: Three dimensional
CT: Computed tomography
